# Adult Cardiac Progenitor Cell Aggregates Exhibit Survival Benefit Both *In Vitro* and *In Vivo*

**DOI:** 10.1371/journal.pone.0050491

**Published:** 2012-11-30

**Authors:** Michael Bauer, Lifeng Kang, Yiling Qiu, Jinhui Wu, Michelle Peng, Howard H. Chen, Gulden Camci-Unal, Ahmad F. Bayomy, David E. Sosnovik, Ali Khademhosseini, Ronglih Liao

**Affiliations:** 1 Division of Cardiology and Division of Genetics, Department of Medicine, Brigham and Women’s Hospital, Harvard Medical School, Boston, Massachusetts, United States of America; 2 Center for Biomedical Engineering, Department of Medicine, Brigham and Women’s Hospital, Harvard Medical School, Cambridge, Massachusetts, United States of America; 3 Harvard-MIT, Division of Health Sciences and Technology, Massachusetts Institute of Technology, Cambridge, Massachusetts, United States of America; 4 Department of Pharmacy, National University of Singapore, Singapore, Singapore; 5 School of Life Science, Nanjing University, Nanjing, China; 6 Martinos Center for Biomedical Imaging, Department of Radiology, Massachusetts General Hospital, Harvard Medical School, Boston, Massachusetts, United States of America; 7 Department of Orthopaedics and Sports Medicine, University of Washington, Seattle, Washington, United States of America; 8 Wyss Institute for Biologically Inspired Engineering, Harvard University, Boston, Massachusetts, United States of America; 9 Harvard Stem Cell Institute, Harvard University, Cambridge, Massachusetts, United States of America; Ohio State University, United States of America

## Abstract

**Background:**

A major hurdle in the use of exogenous stems cells for therapeutic regeneration of injured myocardium remains the poor survival of implanted cells. To date, the delivery of stem cells into myocardium has largely focused on implantation of cell suspensions.

**Methodology and Principal Findings:**

We hypothesize that delivering progenitor cells in an aggregate form would serve to mimic the endogenous state with proper cell-cell contact, and may aid the survival of implanted cells. Microwell methodologies allow for the culture of homogenous 3D cell aggregates, thereby allowing cell-cell contact. In this study, we find that the culture of cardiac progenitor cells in a 3D cell aggregate augments cell survival and protects against cellular toxins and stressors, including hydrogen peroxide and anoxia/reoxygenation induced cell death. Moreover, using a murine model of cardiac ischemia-reperfusion injury, we find that delivery of cardiac progenitor cells in the form of 3D aggregates improved *in vivo* survival of implanted cells.

**Conclusion:**

Collectively, our data support the notion that growth in 3D cellular systems and maintenance of cell-cell contact improves exogenous cell survival following delivery into myocardium. These approaches may serve as a strategy to improve cardiovascular cell-based therapies.

## Introduction

Heart failure is a clinical condition arising from the progressive loss of functional muscle cells following cardiac injury [1]. While it has recently been established that the adult heart possess a progenitor cell population capable of differentiation into functional cardiomyocytes, this capacity for regeneration is severely limited and is unable to adequately account for the lost tissue [2]. The degree of cardiomyocyte loss is closely related to subsequent cardiac dysfunction, as well as cardiovascular morbidity and mortality [1], [3]. Currently, while pharmacologic therapies represent the standard treatment approach for heart failure, these treatment options fail to address the loss of functional cardiomyocytes. Recently, stem-cell based therapies have been explored as a means for regeneration of heart tissue [4], with functional differentiation of implanted stem cells into mature cardiomyocytes [5], [6], [7], [8]. Thus far, different populations of adult stem cells have been examined following implantation in animal models of and humans with heart disease [9], [10], [11], [12], [13]. While the reported therapeutic benefits have been inconsistent, autologous cell implantation has generally been deemed safe. A major obstacle to realizing therapeutic regeneration is the very poor survival of implanted cells [14], [15], [16], [17]. Following implantation, it has been reported that greater than 95% cells die within hours [18], leaving only a small percentage of viable cells. Augmenting implanted cell survival would allow for greater new cardiomyocyte formation and presumably, greater improvement in cardiac performance. The low survival of implanted cells has been attributed to cell death induced by the hostile environment within injured myocardium [15], [19]. Thus far, attempts to overcome this survival obstacle have focused on biochemical means, including but not limited to, preconditioning of cells prior to implantation, exposure to pro-survival factors, and genetic modification of cells [20], [21]. Few reports, however, have examined the importance of biophysical properties, such as cell-cell contact, in modulating implanted stem cell survival.

Similar to the resident stem cells found in other organs, adult cardiac stem cells reside in a microenvironment, or cellular niche, in the myocardium [22], [23], [24], [25]. The cells within the niche are in close contact with each other, and include progenitor cells, cardiomyocytes and surrounding matrix proteins [23], [25]. It has been shown that the establishment of cell-cell contact is beneficial for promoting cell survival [26], [27]. Thus, we hypothesized that delivering cells in 3D aggregates to maintain cell-cell contact may promote implanted cell survival. In this study, utilizing our recently established microwell array methodology, we demonstrated that cardiac side population (CSP) progenitor cells [28], [29], when delivered in 3D cell aggregates exhibit enhanced survival against stressors and toxins *in vitro*, as well as improved survival following implantation *in vivo* in a murine model of cardiac injury.

## Materials and Methods

### Animals

For *in vitro* studies, CSP cells were isolated from eight-week-old male C57/BL6 mice purchased from Jackson Laboratories. Eight-week-old female Friend Virus B-type (FVB) mice were obtained from Charles River Laboratories for *in vivo* ischemia reperfusion and cell implantation experiments. Dual luciferase and GFP transgenic (L2G) mice were kindly provided by Dr. Joseph Wu (Stanford University School of Medicine) [30]. All animal studies strictly adhered to the guidelines of the Harvard Medical School Institutional Animal Care and Use Committee (IACUC) and the National Society for Medical Research. All animal studies were conducted according to guidelines provided by National Research Council, National Institutes of Health and Institute of Laboratory Animal Resources. The protocols were reviewed and approved by the IACUC of Harvard Medical School (protocol number: 04745) or Massachusetts General Hospital (Protocol number: 2011N000009).

### Cardiac Side Population Cell Isolation and Culture

CSP cells were isolated as described previously [28], [29], [31], [32]. Briefly, hearts from adult mice as described above were digested using dispase and collagenase B (Roche, Indianapolis, IN). The mononuclear cell fraction was isolated and stained using 5 µg/ml Hoechst 33342 (Sigma-Aldrich, St. Louis, MO) at 37°C for 90 minutes and further stained for CD31 and Sca-1 (BD, Franklin Lakes, NJ). CD31− Sca-1+ CSP cells were isolated using fluorescence activated cell sorting (FACS Aria, BD, Franklin Lakes, NJ) and cultured in our established proliferation media as described before [28], [29], [31], [32]. The CSP cells used in this study were passage 4–6 cells.

### Microwell Assembly

Microwells at a diameter of 200 µm were generated using micromolding on UV-photocrosslinkable polyethylene glycol dimethacrylate (PEGDM, MW = 1000, 10% in PBS) (Monomer-Polymer & Dajac Labs, Trevose, PA) with 0.5% photoinitiator 2-hydroxy-1-[4-(hydroxyethoxy)phenyl]-2-methyl-L-propanone (Irgacure D2959, Ciba Specialty Chemicals Inc., Florham Park, NJ). To create surfaces capable of PEG attachment, glass slides were treated with 3-(trimethoxysilyl) propylmethacrylate (TMSPMA) (Sigma-Aldrich Co., St. Louis, MO) at 80°C for 12 hours. A patterned PDMS stamp was placed on an evenly distributed film of PEGDM macromer solution on the TMSPMA coated glass slide and photo-crosslinked (λ = 350–500 nm, 180 sec, 10 mW/cm^2^; OmniCure Series 2000 curing station, EXFO, Mississauga, Canada). For adhesive (glass bottom) microwells, the patterned PDMS stamp was first placed on the glass slide and then PEG monomer solution was added at the edge of PDMS stamp. After polymerization the PDMS stamp was peeled from the substrate.

### Generation of CSP Aggregates

**Figure 1:**
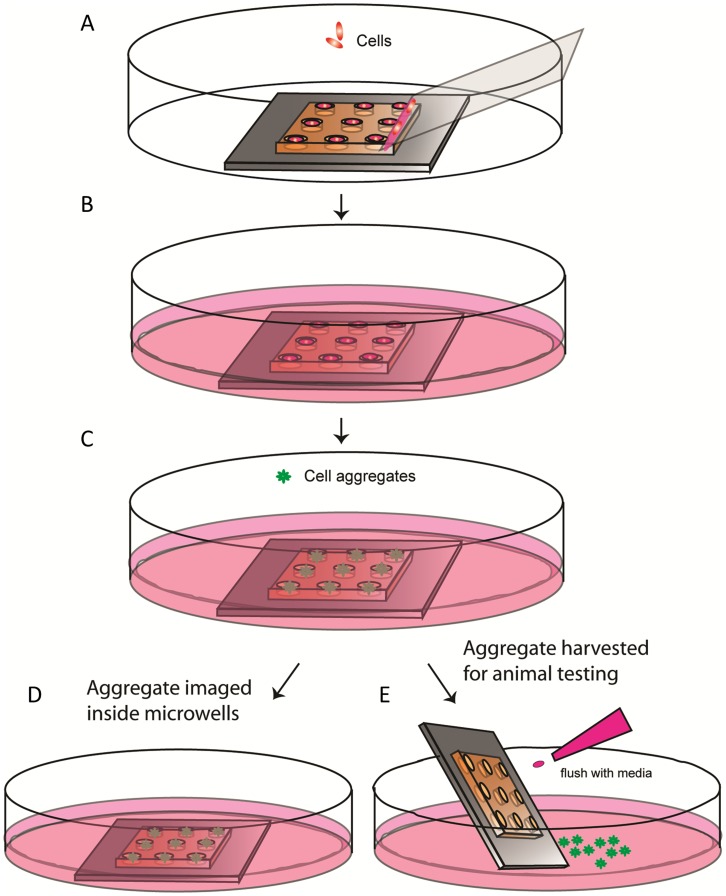
Cell patterning and aggregate formation inside microwells. **A)** Cell patterning. Cells were localized inside the microwells. **B)** After cell seeding, the cells in the microwell array were cultured in a petri dish and aggregates formed within 24 h. **C)** Once the aggregate formation is complete inside the microwells, they can be stained. **D)** Aggregates can be imaged inside microwells. **E)** Aggregates can be easily released from the microwells by gentle flushing with media for other applications.

For controlled cell aggregation, CSP cells were seeded into microwells by using a previously developed method [33]. A schematic summarizing the protocol is shown in **Figure 1**. Briefly, 15 µl of cell suspension (4−16×10^6^ cells/mL) was pipetted along the edge of a cover glass, which was then slowly (∼1 mm/s) wiped across a microwell array. Once the array was traversed, the excess fluid outside the array was removed and the array was immersed in media in a culture dish. After 3D cellular aggregates formed overnight, they were imaged directly inside microwells or harvested by flushing with a stream of culture media.

As a comparison, cell aggregates were also generated by plating cells (2000 cells/well) in 96 well plates (2000 cells/well) with ultra low attachment (ULA) surface (Corning life sciences, Lowell, MA). Twenty-four hours post-seeding, the aggregated size was determined by imaging cells using phase contrast microscopy.

Conventional 2D CSP cell culture was achieved by seeding cells with 5.9×10^3^ cells/mm^2^ using the method similar to the 3D cell aggregate but with glass-bottom microwells. Cells were cultured overnight in their respective conditions for all *in vitro* and *in vitro* experiments.

### *In vitro* Viability Assays

Cell viability *in vitro* was determined using direct fluorescence microscopy imaging methods and FACS-based analysis. For imaging, CSP cells in either 3D aggregates or 2D culture were stained using a combination of ethidium homodimer (EthD) (Invitrogen, Carlsbad, CA) and DAPI (Invitrogen, Carlsbad, CA). The microwells were washed with PBS and incubated with EthD (4 µM) for 10 minutes at 37°C to label dead cells. Cells were washed using PBS, fixed with 4% paraformaldehyde and counter-stained with DAPI (5 µg/ml) for 10 minutes at 37°C and washed with PBS. To quantify cell viability, microarrays were imaged using a fluorescence microscope equipped with a motorized stage (Axiovert 200M, Zeiss, Thornwood, NY). Fluorescence intensities for both DAPI and EthD were measured using ImageJ (NIH, Bethesda, MD). Briefly a grid of ROIs was produced to match the microarray and then fluorescence values in both the DAPI and the EthD channels were measured. Minimal values in each ROI were taken as the local background and subtracted from the fluorescence reading. The cell death was calculated as the ratio between EthD and DAPI readings.

For FACS-based cell death analysis, cells in microwells were incubated with EthD as described above. Following staining, the cell aggregates or 2D cultures were washed out and dissociated into single cell suspensions with 0.05% trypsin (Gibco, Invitrogen, Carlsbad, CA). Cell death analysis was performed using flow cytometry (Accuri C6, Accuri Flow Cytometry, MI).

### Hydrogen Peroxide Treatment and Anoxia/Reoxygenation

To determine the susceptibility to stress, CSP cells in cell aggregates or 2D cultures were treated with 200 µM H_2_O_2_ or anoxia followed by reoxygenation. Briefly, CSP cells were incubated with culture medium containing 200 µM H_2_O_2_ or vehicle for 2 hours at 37°C. For the anoxia and reoxygenation, cells were subjected to 24 hours anoxia, 5% CO_2_ balanced medical grade 99.9999% N_2_ (Airgas, Salem, NH), using a standard hypoxia chamber followed by 2 hours reoxygenation. Cells cultured under regular culture conditions for the same period of time were used as control. Viability of cells was determined as described above.

### Mouse Model of Ischemia Reperfusion and Cell Implantation

A murine model of ischemia reperfusion was generated by ligating the left coronary artery of FVB mice for 45 minutes, followed by release, as described previously [34]. Two weeks following ischemia reperfusion, 1×10^5^ CSP cells in 12.5 µl total volume in either 3D aggregate or single cell suspensions were delivered via direct myocardial injection into the border zone of the infarct [34].

### *In vivo* Cell Survival Assay

Implanted cell survival was determined by bioluminescence imaging *in vivo* and immunohistochemtry *ex vivo*. For *in vivo* imaging, the mice were anesthetized with isoflurane and an aqueous solution of the substrate D-Luciferin (Molecular Imaging Products, Bend OR) was injected intraperitoneally (200 mg/kg body weight at 15 mg/ml). The animals were then allowed to recover before being re-anesthetized and placed in the Xenogen IVIS Spectrum imaging system (Caliper Life Sciences, Hopkinton, MA) with the platform preheated to 38°C. The CCD (Charge-coupled device) camera was cooled to −90°C and the field of view (FOV) set to 22.4×22.4 cm^2^. Luminescent images were acquired with a 300 s exposure time, 8 binning, 1 f/stop, open filter, and with a spatial resolution of 270 µm. Photographic images of the subjects were acquired as well.

Quantification of the luminescence signal was performed using the Living Image software (Caliper) by applying a region of interest (ROI) over the heart and measuring the photon intensity in average radiance (photons·sec^−1^·cm^−2^·sr^−1^). Measurements were taken at 0, 4, and 6 days post cell implantation, background subtracted, normalized to the corresponding day 0 signal, and expressed as percent survival. Each data point represents n = 6.

### Data Analysis and Statistics

Data were collected using a spreadsheet program and further analyzed using the R statistical framework 11. Student’s t-tests were used to compare two groups, while analysis of variance (ANOVA) to compare three or more groups. A p<0.05 was considered statistically significant. Data are presented as mean ± S.D.

## Results

### Size of CSP Cell Aggregates is Regulated by Controlling Cell Seeding Density in Microwells

**Figure 2:**
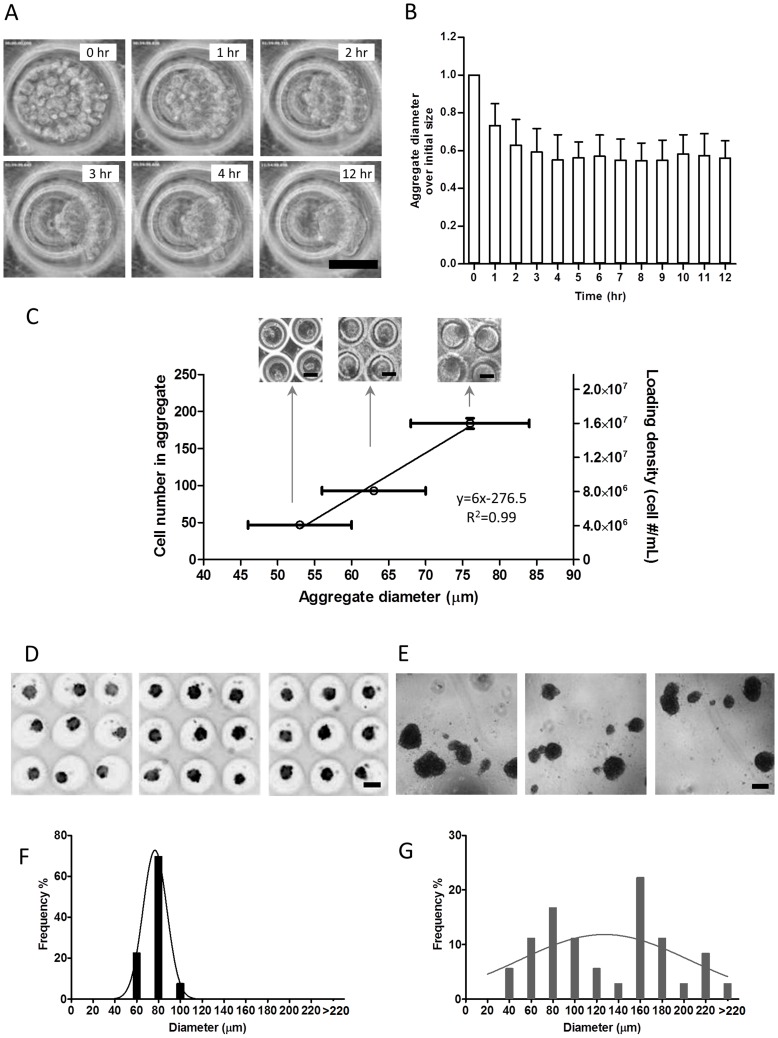
Characterization of aggregate formation and uniformity. **A)** Time course of aggregate formation in the microwell at 0,1,2,3,4, and 12 h post seeding. **B)** Quantification of aggregates size over time (n = 2). **C)** Size of the aggregates can be controlled by seeding microwell arrays for CSP aggregation with different cell numbers in microwells. The cell suspension densities used for seeding were 4×10^6^/mL, 8×10^6^/mL, and 16×10^6^/mL, resulting in aggregates with controllable sizes defined hereinafter as small aggregate (S), medium aggregate (M), and large aggregates (L), respectively. **D)** Subsets of a microwell array with CSP cell aggregates after 24 h showing high uniformity. **E)** CSP cells cultured in an ultra low adhesive (ULA) 96-well plate for 24 h. Cells form aggregates of various sizes. **F)** Frequency distribution of diameter of aggregates formed in microwells shows narrow distribution. The standard deviation (S.D.) of Gaussian distribution fitting is 10.9 µm. **G)** Frequency distribution of diameter of aggregates formed in ULA 96-well plates shows wide distribution. The S.D. of Gaussian distribution fitting is 77.7 µm. (All bars represent 100 µm).

The formation of CSP cell aggregates took place in culture within a short period of time. CSP cells seeded in microwells and imaged using a time-lapse mode in phase-contrast showed initial formation of aggregates within 1 hour post seeding (**Figures 2A** **)**. Aggregate size reduced to the final size in approximately 3 hours (**Figures 2A** **)**. Thereafter, over a subsequent 9 hours, aggregate size did not significantly increase but a further smoothening of the surface was observed (**Video S1**).

To determine whether the cell aggregate size can be regulated, we seeded cells in three concentrations ranging from 4×10^6^, 8×10^6^, to 16×10^6^ CSP cells per mL in microwell arrays. We found that the number of cells in each aggregate increased with greater seeding density as measured 24 hours following seeding (**Figure 2B**). Moreover, the diameter of cell aggregate correlated (R^2^ = 0.99) to the cell number in each aggregate (**Figure 2C**). We next compared the uniformity of size of each aggregate between cell seeding in microwells and conventional 96 well non-adherent plates with ULA surface. Twenty-four hours following seeding, CSP cells cultured in suspension formed aggregates in both microwells (**Figure 2D**) and ULA 96 well plates (**Figure 2E**). As shown in **Figure 2F**, the size of CSP cells was homogenous with low variation with standard deviation of 10.9 µm. In contrast, the aggregate size of cells cultured in ULA 96 well plates varied largely with broader range of distribution as evidenced by a 77.7 µm standard deviation (**Figure 2G**). These data demonstrated that microwell methodology can be used to generate desired aggregate size for biological applications.

### CSP Cell Aggregates are Less Susceptible to Oxidative and Hypoxic Stress

**Figure 3:**
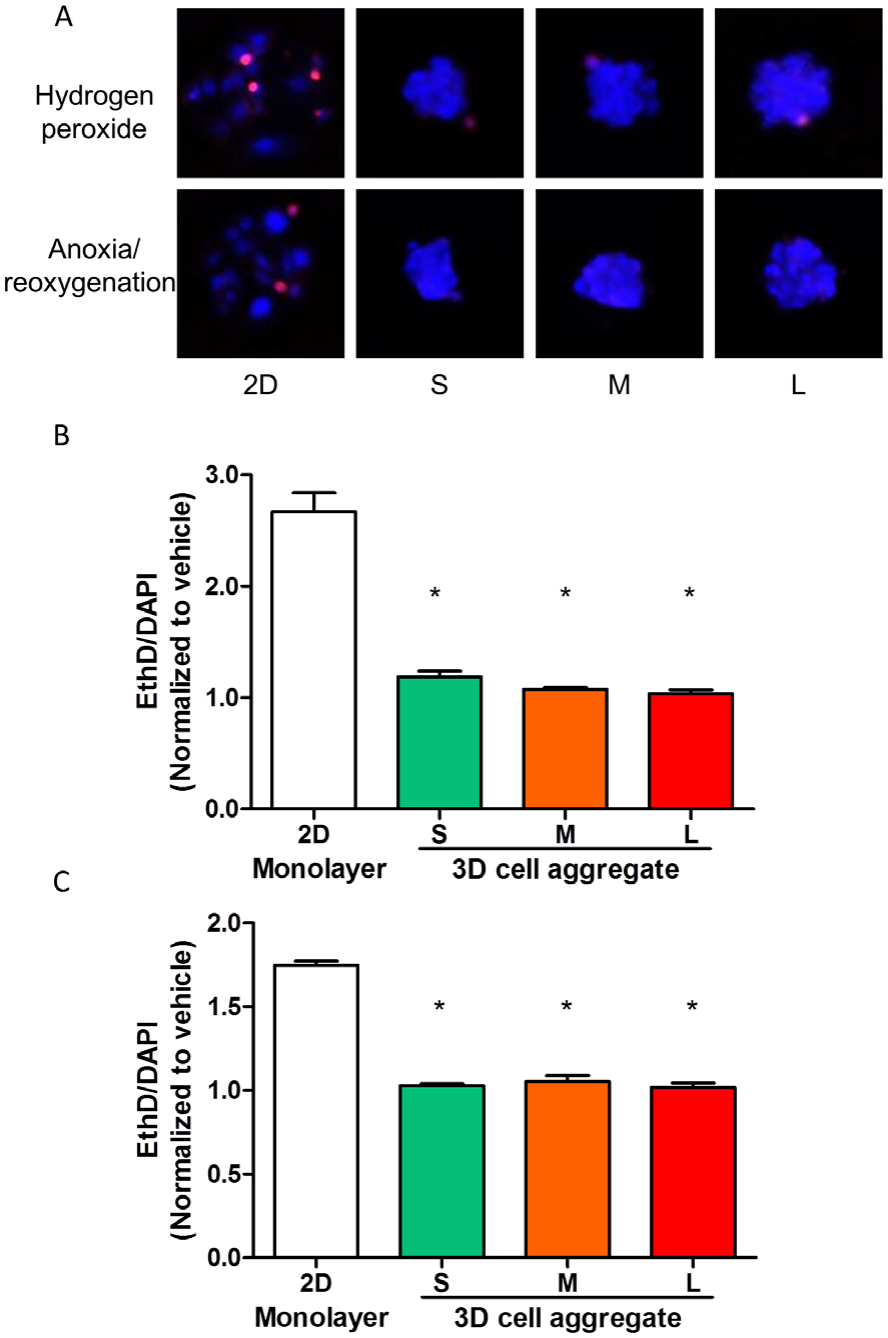
Aggregate survival tests *in vitro*. **A)** Subsets of microwell arrays with 2D monolayer of cell culture (2D) and aggregates of three sizes (S, M, and L). Hydrogen peroxide and anoxia/reoxygenation treatments were employed to induce cell death. EthD (red) and DAPI (blue) staining were performed for the determination of cell death. **B)** Quantification of dead CSP cells in 2D single layer culture and aggregates with variable diameters subjected to 200 µM-hydrogen peroxide treatment using EthD/DAPI fluorescent intensity ratio. Data were normalized to the vehicle groups of 2D monolayer culture and aggregates in three sizes. **C)** Quantification of dead CSP cells in 2D single layer culture and aggregates with variable diameters subjected to anoxia/reoxygenation using EthD/DAPI fluorescent intensity ratio. Data were normalized to the vehicle groups of 2D monolayer culture and aggregates in three sizes.

To test the hypothesis that cells in 3D aggregates enhance protection against stressors and toxins, CSP cells in either 2D monolayer culture or 3D cell aggregate with aggregates of three different sizes, S: 53±7 µm; M: 63±7 µm; L: 76±8 µm, were subjected to 200 µM H_2_O_2_ for 2 hours to mimic the increased oxidative stress present in injured myocardium. Cells grown in 3D cell aggregates were less susceptible to increased oxidative stress compared to those cultured in 2D monolayer as determined by EthD/DAPI ratio. As demonstrated in **Figure 3A and 3B**, cells cultured as 3D aggregates showed a lower EthD/DAPI ratio as compared to cells in single layer. Interestingly, this survival benefit was independent of the cell number per aggregate within the range tested in this study (47±1 to 184±7 cells). In addition, we examined the response of CSP cells to 24 hours of anoxia followed by 2 hours reoxygenation. Similar to results obtained following H_2_O_2_ exposure, reduced cell death, as determined by EthD/DAPI, was observed in 3D cell aggregates (**Figure 3C and 3D**, p<0.05, n = 3). These observations were confirmed using additional measures of cell death, including by FACS analysis, in a subset of experiments (**Figure S1A** and **S1B**).

To verify that protection from cell death observed in cell aggregates was not due to the in-accessibility of dye staining in 3D aggregate format, we subjected CSP cell aggregates to supraphysiologic concentrations of H_2_O_2_ and were indeed able to detect dead cells using the fluorometric assay (**Figure S1C)**.

### CSP Cell Aggregates Show Improved Survival *in vivo*

**Figure 4:**
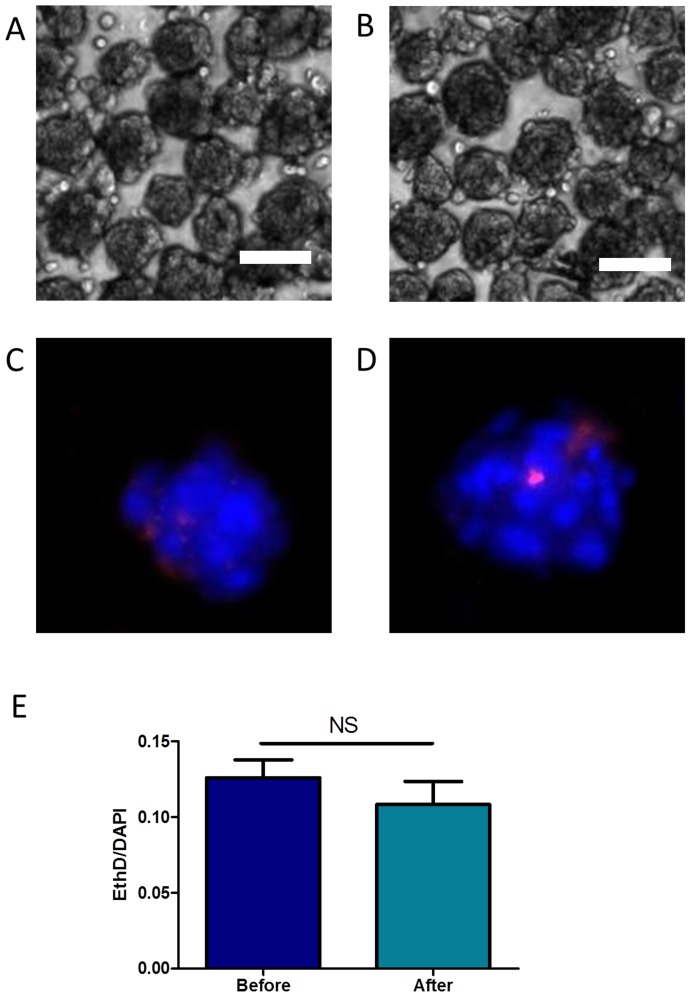
Aggregate integrity and survival in fluidic manipulations. **A)** Aggregates formed in microwells can be easily flushed out from the microwell and centrifuged while remaining intact. **B)** Aggregate can be easily passed through a 30G needle without loosing integrity. **C)** A representative DAPI/EthD fluorescent image of aggregates before injection. **D)** A representative DAPI/EthD fluorescent image of aggregates after injection. **E)** Quantification of dead CSP cells in aggregates passing a 30G needle using EthD/DAPI fluorescent intensity ratio. (All bars represent 100 µm).

Prior to testing the survival of cell aggregates *in vivo*, we sought to determine whether CSP cell aggregates were able to withstand the shear stress of passing though a 30G needle, which is required for intra-myocardial implantation. As shown in **Figures 4A & 4B**, the gross morphology of CSP cell aggregates was unchanged following passage through a small caliber needle. Moreover, cell viability, as determined by cell death assay, was unchanged with passage (**Figures 4C** – **4E**).

**Figure 5:**
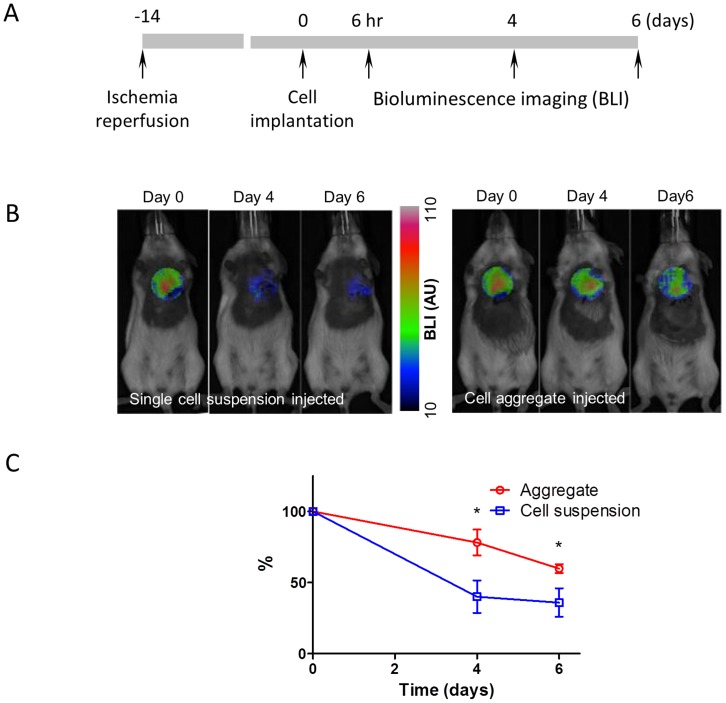
CSP cell survival *in vivo* following cardiac injury. **A)** Protocol to measure the *in vivo* survival of CSP aggregates and suspensions. **B)** Representative serial bioluminescence images (BLI) of mice injected with CSP cell aggregates and CSP single cell suspensions. **C)** Percentage of CSP cell survival measured with BLI.

A myocardial ischemia-reperfusion (post-IR) model was used to assess the survival benefit of CSP cell aggregates *in vivo*. CSP cells were isolated and cultured from mice over expressing luciferase/eGFP (L2G mice). Two weeks post-IR, 100,000 L2G CSP cells in either single cell suspension or 3D aggregates (76±8 µm) were injected into the border zone. The initial graft survival was determined 6 hours post injection as shown in the **Figures 5A & 5B**, with a marked initial cell engraftment. Only 40% of implanted cells remained alive at day 4 post implantation when cells were delivered in single cell suspension, whereas approximately 80% of implanted cells survived when cells were delivered as cell aggregates (**Figure 5C**). CSP cells in either aggregates or suspension showed clear bioluminescence signals 6 hours post injection (**Figure 5B** **)**, and the survival benefit of 3D cell aggregates persisted at 6 days post-cell implantation (**Figure 5C**). Taken together, these data support the hypothesis that cells delivered in the form of 3D aggregates exhibit greater survival when implanted *in vivo* following cardiac injury.

## Discussion

In this report, we utilized a microwell array system to study the effect of 3D cell culture on cardiac side population (CSP) cells, a proven adult resident cardiac progenitor cell population [24], [28]. Using this newly developed system, CSP cell survival both *in vitro* and *in vivo* was remarkably improved in 3D aggregates. This work suggests that manipulation of cellular aggregates and growth of cells in 3D structures may facilitate cell-cell interaction and provide strategies for enhancing therapeutic tissue regeneration.

### Microwell Approach is Feasible for Regulating Size of Cell Aggregate

Similar to other type of cells, CSP cells form aggregates when cultured in suspension. However these aggregates vary in size and the numbers of aggregates are not easily controlled. Results from embryonic stem cells have suggested an influence of aggregate size on biologic properties [35]. Furthermore, the ability to control the size of cell aggregates allows for closer replication of conditions believed to exist *in vivo* in the cellular niche. There are several methods that have been previously used to control aggregate size when forming aggregates. Most common among these is the hanging drop method [36]. However this method does not allow for the production of large numbers of aggregates for mass production. Microwells can be used to control aggregate size and contrary to hanging drops, they facilitate the production of a large number of aggregates [37]. When seeded into microwells, CSP cells formed homogenous aggregates. Importantly, the size of the aggregate was tightly dictated by the number of cells seeded into each well. The benefits of microwells also include direct *in vitro* testing within wells without having to transfer cells. Furthermore, harvesting aggregates from microwells is simple and straightforward. This application may be adapted for seeding stem or progenitor cells from different sources for experimental or therapeutic applications.

### Survival Benefit of 3D Cell Aggregate to Stress or Injury *in vitro and in vivo*

Cell therapy aims at regenerating lost myocardium by delivering primitive cells into the injured hearts. While some functional benefits have been observed in most prior studies of cell implantation, to date these improvements have been relatively modest, and ascribed to the paracrine effects of implanted cells rather the direct differentiation of engrafted cells. One major reason remaining for the lack of significant tissue regeneration is the poor survival of implanted cells. Toward this end, several strategies have been explored to augment graft survival [15] in the past. Most of the efforts have been focused on supplementing cells with soluble survival or anti-apoptotic factors [38], overexpression of proteins involved in survival pathways [39], as well as delivery of a cocktail of miRNA species [40]. Limited efforts, however, have been put forth to exploit the intrinsic properties of 3D environment of cells within tissue. Using microwell arrays as an *in vitro* test platform we compared the viability of CSP cells cultured in aggregates to CSP cells cultured as 2D monolayer under oxidative stress. To account for possible effects of cell density or cell number, 2D monolayer controls were performed in microwells with adhesive bottom. It is noteworthy that while the combination of Calcein AM and EthD is a well known cell viability stain, this method is not suitable to determine the cell survival under the condition of oxidative stress due to the fact that Calcein itself is a sensor for oxidative activity [41]. Therefore, we modified the method, combining EthD for staining nuclei of necrotic cells with a counterstain of DAPI to stain for total nuclei. This method delivered reliable results when used in fluorometric measurements verified by the FACS analysis.

Our observations are consistent with reports suggesting that 3D cell aggregates exhibit better survival compared to cells cultured in monolayer conditions *in vitro* [27]. In our study, however, the observed effects were not dependent on aggregate size, at least among the three aggregate sizes tested. In contrast, a size dependent effect has been reported in studies of embryonic stem cells aggregates [42]. The differences in cell types and aggregate sizes studied, as well as the specific end points tested in the two studies may underscore the varying results between studies. We extended these observations of survival benefits with 3D cell aggregates, and have now provided evidence suggesting that the benefits of 3D cell aggregates persist following implantation into mouse hearts with ischemia perfusion injury *in vivo*. It is important to note that given aggregate size, the suitability of delivering stem cells via intra-coronary methods needs to be carefully evaluated, particularly as delivery of aggregates has the potential for micro-embolization. Furthermore, future studies will be required to subsequently determine the *in vivo* functional benefits of 3D cell aggregates on progenitor cell differentiation, tissue regeneration, cardiac function and cardiovascular outcomes.

In summary, in this study we provide the first proof of concept of (1) the feasibility to generate 3D cell aggregates with control dimension; (2) that adult cardiac progenitor cells when cultured in 3D aggregates format tolerate oxidative stress and anoxia reoxygenation better *in vitro*; and finally, (3) 3D cell aggregates possess survival benefits when implanted into mouse hearts following ischemia reperfusion, *in vivo*. Collectively, our data provides evidence that these methods may be utilized to advance the efficacy of cell-based therapy in the cardiovascular system.

## Supporting Information

Figure S1 **Quantification method of dead cells and validation using FACS. Cells subjected to 200 µM hydrogen peroxide for 2 hr.** **A)** Representative EthD/DAPI fluorescent microscopic images and quantification of dead cells using EthD/DAPI fluorescent intensity ratio. **B)** Representative FACS profiles and quantification of dead cells in FACS. The X-axis represents forward scatter (FSC) and y-axis EthD fluorescence in channel 3 (fl-3). **C)** CSP cell aggregates subject to supra-physiologic concentrations of hydrogen peroxide.
(TIF)

Video S1 **A video clip shows the time course of CSP cell aggregation in a microwell.**
(MP4)
