# Supplementary figures and images for: Adult Cardiac Progenitor Cell Aggregates Exhibit Survival Benefit Both In Vitro and In Vivo
Source: PLoS One. 2012 Nov 30;7(11):e50491. doi: 10.1371/journal.pone.0050491 (PMC3511575; doi:10.1371/journal.pone.0050491)

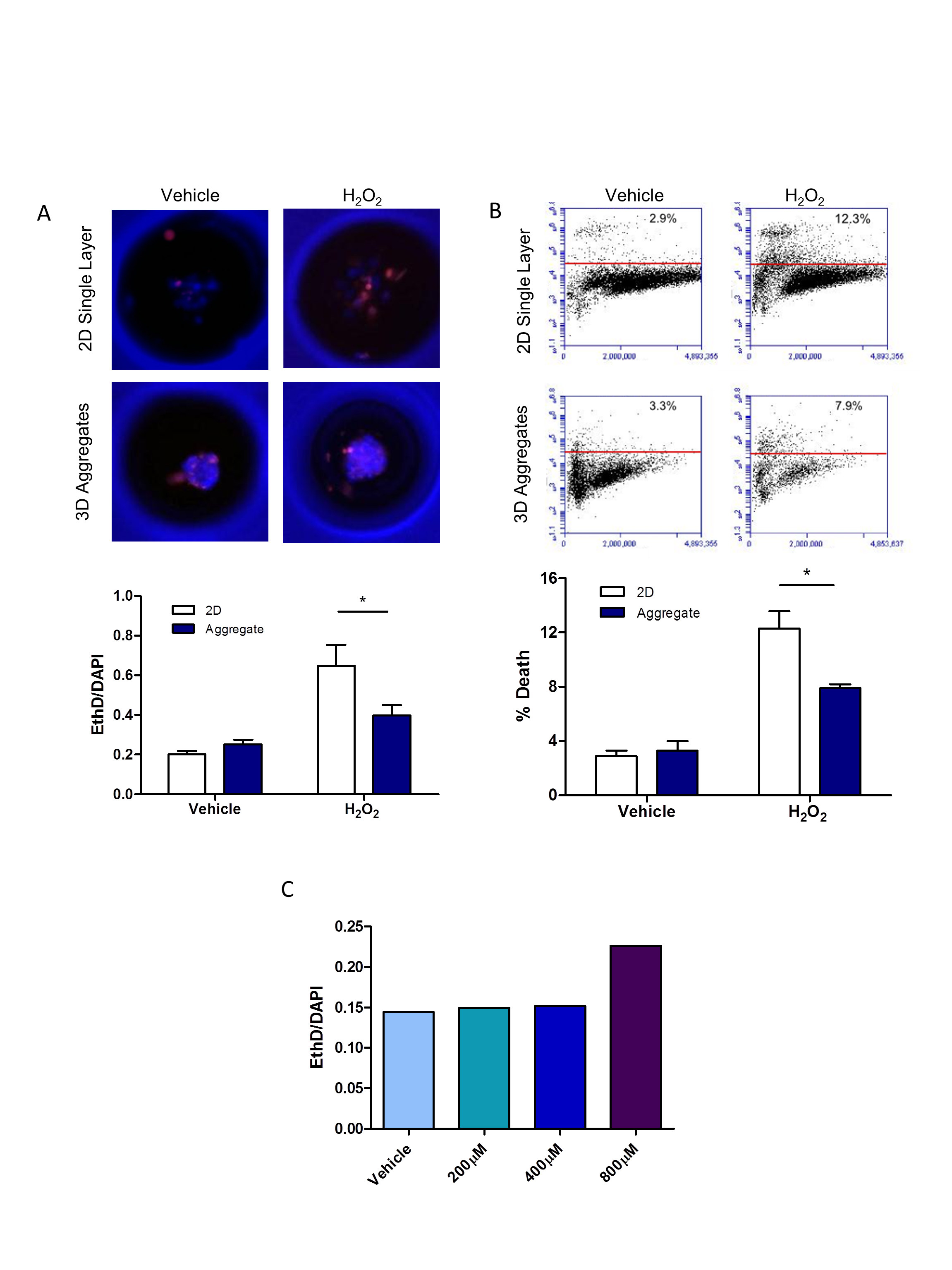

Supplement: Figure S1 — Quantification method of dead cells and validation using FACS. Cells subjected to 200 µM hydrogen peroxide for 2 hr. A) Representative EthD/DAPI fluorescent microscopic images and quantification of dead cells using EthD/DAPI fluorescent intensity ratio. B) Representative FACS profiles and quantification of dead cells in FACS. The X-axis represents forward scatter (FSC) and y-axis EthD fluorescence in channel 3 (fl-3). C) CSP cell aggregates subject to supra-physiologic concentrations of hydrogen peroxide. (TIF) [file pone.0050491.s001.tif]
